# Expanding Soil Invertebrate Knowledge in Panama: The Genus *Lepidocyrtus* (Collembola, Entomobryidae) in the Parque Natural Metropolitano as a Study Case

**DOI:** 10.3390/insects15120951

**Published:** 2024-11-30

**Authors:** Alba Enguídanos García, Carles Galià-Camps, Claudia Massiel Pérez-González, Dionora Víquez, Eduardo Mateos

**Affiliations:** 1 Department of Evolutionary Biology, Ecology and Environmental Sciences, Universitat de Barcelona, 08028 Barcelona, Spain; emateos@ub.edu; 2 Biodiversity Research Institute (IRBio), University of Barcelona, 08028 Barcelona, Spain; 3 Blanes Center for Advanced Studies (CEAB-CSIC), Access Cala St. Francesc 14, 17300 Blanes, Spain; 4 Universidad de Panamá, Departamento de Genética y Biología Molecular, Facultad de Ciencias Naturales Exactas y Tecnología, Panama 0819-07289, Panama; claudia.perezg@up.ac.pa; 5 Metropolitan Natural Park, XFP3+47H Juan Pablo II Avenue, Panama 0819-07289, Panama; direjecutiva@cwpanama.net

**Keywords:** species complex, molecular barcoding, phylogenetics, taxonomy, pseudopores, dental tubercle

## Abstract

Panama, located in the heart of the Mesoamerican hotspot, harbors an extraordinary species diversity across the Tree of Life. The Collembola species of the genus *Lepidocyrtus* play an important role in soil biological processes such as decomposition, being used to monitor soil health and functional parameters. However, the limitation of morphological characters and molecular resources hampers the evaluation of local soil diversity. Here, using 30 *Lepidocyrtus* specimens collected in the Parque Natural Metropolitano (PNM), we unravel the diversity of this Panamanian protected area through molecular tools and new taxonomic traits. Our phylogenies, in combination with species delimitation analyses, indicate that the PNM harbors an extremely rich community of *Lepidocyrtus* species, two of them cited in Panama for the first time, and three of them potentially new to science. We highlight that the presence of the dental tubercle and pseudopores on the BP4 region are not monophyletic and, therefore, can be used as supplementary characters to morphologically resolve species complexes. Overall, this study sheds light on the *Lepidocyrtus* richness of the PNM, which acts as a shelter for Panamanian and the Mesoamerican hotspot species.

## Simple Summary

Panama, a biodiversity-rich country, is home to many important species that are crucial to maintain ecosystems healthy. This study focused on a group of small soil-dwelling animals, *Lepidocyrtus* springtails, which play a key role in breaking down organic material and indicating soil health. However, it is difficult to identify them accurately due to limited physical traits and genetic data. By analyzing 30 specimens from a protected area in Panama, we discovered a surprising variety of species, including two new records in Panama and three potentially new to science. We also identified new features that could help scientists better distinguish between species. This research highlights the importance of Panama’s natural areas in preserving diverse species critical to the environment.

## 1. Introduction

Collembola (springtails), with more than 9000 species reported worldwide [1], are numerically dominant microarthropods in most soil ecosystems. In these habitats, collembola species play key roles in detrital food webs, modulating litter decomposition processes, and contributing to soil microstructure [2,3].

*Lepidocyrtus* Bourlet, 1839, is the third largest genus of Collembola with ~270 described species and is cosmopolitan in distribution [1]. Recent research on this cosmopolitan genus using both morphological and molecular data has revealed the existence of abundant species complexes in Europe [4,5,6,7] and the Neotropical regions [8,9]. These findings suggest that the real diversity of *Lepidocyrtus* may be vastly underestimated on a global scale.

To unravel the overlooked diversity of the genus, it is necessary to find new morphological characters that allow an unequivocal diagnosis of the species. The number and arrangement of pseudopores on the body and appendages may contribute to this objective [5,10]. Pseudopores are integumentary structures of unknown function described for the first time in Collembola by Gisin (1963) [11]. Despite their interest at different taxonomic levels [10], the knowledge of their presence in the different groups of Collembola is limited. Since the publications of Gisin [11,12], all *Lepidocyrtus* species descriptions mention the 1 + 1 dorsal pseudopores from the second thoracic segment to the fourth abdominal segment and the 2 + 2 (rarely 3 + 3 or 4 + 4) pseudopores on the dorsal manubrial plate. In addition to the dorsal pseudopores found by Gisin, Mateos et al. (2021) [5] described a pattern of pseudopores in different regions of the body in many European species. The number and arrangement of pseudopores in some regions of the body can improve the taxonomic assignment of the *Lepidocyrtus* species and help in phylogenetic studies. Moreover, their presence in the basal plate of the fourth abdominal segment (BP4 sensu Hopkin 1997 [13], p. 71) has been suggested as a character with a potential phylogenetic signal [5]. However, the distribution of pseudopores on the body and appendages in the genus *Lepidocyrtus* are only known from a few European species [5] and the four Neotropical species of the subgenus *Fractocyrtus* (endemic to South America [14]). For this reason, it is necessary to evaluate the number and position of *Lepidocyrtus’* pseudopores at a global scale to evaluate the following: (1) whether these have a phylogenetic signal, and (2) whether they can be used as a fine-scale taxonomy trait to unravel the real diversity hidden within this genus.

Panama is located in the heart of the Mesoamerican biodiversity hotspot, the most species-rich region worldwide. Despite covering only 0.5% of the Earth’s surface, this region is home to approximately 5% to 12% of the planet’s species [15,16,17]. Thus, Panama harbors an extraordinary species diversity across the Tree of Life. Given the country’s exceptional biodiversity and its critical role as a reservoir of species in the context of global climate change, Panama has established an extensive network of protected natural areas. Among these, the Parque Natural Metropolitano (PNM; Metropolitan Natural Park) is a remarkable area, spanning 232 hectares + 1159.43 m^2^ and enclosed by tropical moist and dry forests. It is located on the Pacific side of the country, just a few kilometers from Panama City, and therefore highly influenced by the expansion of the country’s largest urban nucleus, home to over 500,000 inhabitants. The PNM’s proximity to the Panama Canal further enhances its ecological and strategic importance. The park supports a diverse array of tropical biodiversity, with 418 vertebrate species and 633 plant species, making it an exceptional site for tropical biodiversity research. Its protected status fosters rich biological diversity, while the proximity to urban development encourages the presence of anthropogenic species. Furthermore, its location near the Panama Canal presents a valuable opportunity for early detection of introduced species. In this context, the Collembola of the genus *Lepidocyrtus* serve as exemplary models for studying such dynamics, as they occupy a broad range of habitats and ecological niches.

To date, a total of 42 *Lepidocyrtus* species have been reported in the Neotropical region [1,14,18,19,20,21]. From these, only four have been cited in Panama: *L. usitatus* Folsom, 1927, whose type locality is Panama and Honduras [22]; *L. vexans* Denis, 1933, with its type locality in Costa Rica; *L. balteatus* Mari Mutt, 1983 with its type locality in Venezuela [8]; and *L. floridensis* Snider, 1967, with its type locality in Florida [23]. However, the biodiversity of this region is highly understudied, and the applicability of genetic tools, such as the genetic barcode cytochrome oxidase I (*cox1*), in addition to the morphological characters, may provide invaluable knowledge on the real diversity of Panamanian *Lepidocyrtus*.

Here, by conducting a shallow sampling on the Parque Natural Metropolitano, we aim to reveal the extreme *Lepidocyrtus* biodiversity of Panama and characterize new taxonomic traits for their identification. By generating new data from 30 Panamanian individuals, most of them juveniles, we detected a total of six species using the molecular marker *cox1*. Moreover, for those species with adult specimens (*L.* cf. *nigrosetosus* Folsom, 1927, and *L. olena* Christiansen and Bellinger, 1992), we sequenced the molecular markers cytochrome oxidase II (*cox2*) and elongation factor (*EF-1α*), usually used on *Lepidocyrtus* studies, to place them in a phylogenetic framework. Finally, we expand the set of morphological characters in the Neotropical species of the genus *Lepidocyrtus* by describing the quantity and arrangement of pseudopores in the body and appendages of two of the species found in the study area. Overall, this study contributes to the knowledge of the springtail biodiversity of the genus *Lepidocyrtus* from the PNM, evidencing the importance of this protected region for the Panamanian and Mesoamerican biota.

## 2. Materials and Methods

### 2.1. Study Area and Taxon Sampling

**Figure 1:**
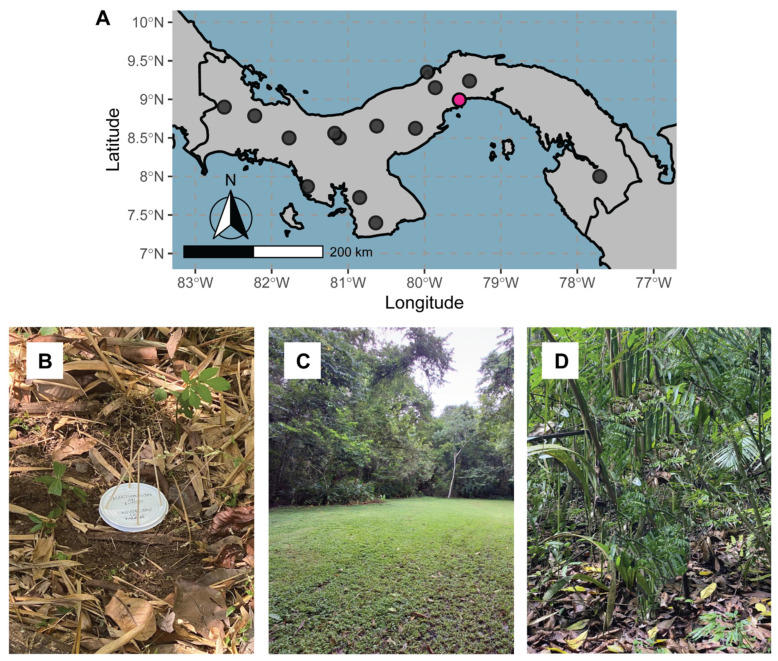
Sampling localities of *Lepidocyrtus*. (**A**) Map of Panama and the historical sampling points for the genus *Lepidocyrtus* [8]. In pink color, we highlighted the new location sampled in the present study. (**B**) Pitfall trap settled at the floor level. Note that the lid is elevated a few millimeters to allow the entrance of small organisms while avoiding larger species and the entrance of rainfall. (**C**) Detail of the area of plot #1, situated in a grassy area. (**D**) Detail of the area of plot #2, situated below a high canopy within the jungle forest.

We collected collembola specimens in the Parque Natural Metropolitano (8°59′31.5″ N, 79°32′38.7″ W), near the bunker El Castillo, by placing a total of eight pitfall traps at the floor level (Figure 1A). Since most members of *Lepidocyrtus* live under leaf litter or at the base of grasses and other vegetation, pitfall traps are a very effective technique for collecting and studying them (Figure 1B). Four pitfalls were placed in a human-perturbed grassy extension (Figure 1C), whereas the four remaining ones were placed in a high-canopy jungle forest with abundant leaf litter (Figure 1D). These two plots were separated by ~200 m. The pitfalls were settled on 28 January 2024, left for a whole week, and collected on 4 February 2024, to optimize time and precipitation probability. We preserved all collected specimens from the pitfalls in 96% ethanol. In the lab, we sorted out all collembola from each pitfall, providing each with its sample code, and assigned them to a species based on their external traits, providing each of the individuals with a specimen code (Table 1). From the 30 *Lepidocyrtus* specimens we collected, 15 were studied only morphologically, 10 were studied both morphologically and molecularly, and 5 were studied only on a molecular level as they were very small.

### 2.2. DNA Extraction and Sequencing

We extracted DNA from 15 specimens, using their entire bodies (without the head, as it was kept for morphological purposes), with the EZNA Tissue DNA Kit (Omega Bio-tek, Norcross, GA, USA), following the manufacturer’s protocol. After concluding an overnight digestion step, we recovered the specimens’ cuticles and kept them as vouchers. We amplified the fragment of the mitochondrial gene that encodes for the first half of the cytochrome oxidase I subunit (*cox1*) region, corresponding to the standard animal DNA barcode (C1-J-1490: 5′-GGT CAA CAA ATC ATA AAG ATA TTG G-3′; C1-N-2198: 5′-TAA ACT TCA GGG TGA CCA AAA AAT CA-3′) [24]; a fragment of the mitochondrial gene cytochrome oxidase subunit II (*cox2*) (tRNA-K-LcuJ: 5′-GAG CGT ATT ATA AAG CGG TTT AAG-3′; tRNA-L-LcuN: 5′-CAG ACT AGT GCC ATG AAT TTA AGC-3′) [25]; and a fragment of the elongation factor-1 alpha (*EF-1α*) nuclear gene (EFLcuJ: 5′-ATG GGG GCA AGA TAG CGT CAA-3′; EFLcuN: 5′-TGA AGG CTG AAC GTG AAC GTG G-3′) [25]. We conducted polymerase chain reactions (PCR) for amplification at a final volume of 20 μL mixes, which included 10.8 μL of distilled H_2_O, 5 μL of MyRed buffer (Bioline Inc., London, UK), 0.2 μL of MyRed Taq DNA polymerase (Bioline Inc., London, UK), 0.5 μL of respective primers at 10 nM, and 3 μL of DNA. Thermocycling consisted, for the *cox1* gene, of an initial denaturation step at 94 °C for 5 min, followed by 35 cycles at 94 °C for 30 s, 42 °C for 35 s, and 72 °C for 45 s, with a final extension step that lasted 5 min at 72 °C; for the *cox2*, the initial denaturation step was 94 °C for 5 min, followed by 35 cycles at 94 °C for 30 s, 48 °C for 35 s, and 72 °C for 45 s, with a final extension step that lasted 5 min at 72 °C. For the nuclear *EF-1α* gene, it consisted of 5 min at 98 °C, followed by 35 cycles at 94 °C for 30 s, 55 °C for 35 s, and 72 °C for 45 s, with a final extension step of 5 min at 72 °C. Positive amplicons were purified using the Exo-SAP-IT (Thermo Fisher Scientific Baltics UAB, Vilnius, Lithuania), and the purified PCR products were sequenced in both directions at Macrogen Inc. (Madrid, Spain). We used the same primers for both PCR and sequencing.

For the three genes, we edited and assembled forward and reverse raw sequences in Geneious Prime 2022.0.1 (Biomatters, Auckland, New Zealand) [26]. Moreover, we downloaded additional *Lepidocyrtus* sequences from the NCBI (National Center of Biotechnology Information) public database [27], along with sequences of three Panamanian specimens of the genus *Seira* to be used as an outgroup (Table 2). Each molecular marker’s sequence was aligned with the MAFFT [28] plugin implemented in Geneious, using the G-INS-i algorithm. Sequences were translated to amino acids to rule out the presence of stop codons. We visually inspected all alignments and curated them when necessary by performing minor manual adjustments. We built four matrices for downstream analyses: *cox1*, *cox2*, *EF-1α*, and the concatenated *cox2* + *EF-1α* matrix. All the matrices included three Panamanian specimens of the genus *Seira* as outgroups. We only concatenated the *cox2* and *EF-1α* matrices because, in collembola, previous research using the *cox1* had exclusively barcoding purposes, while studies using *cox2* and *EF-1α* aimed for phylogenetic approaches. Thus, individuals previously analyzed with *cox1* had no available sequences for *cox2* and *EF-1α*, and vice-versa.

### 2.3. Phylogenetic Analyses

We conducted phylogenetic analyses under a maximum likelihood (ML) approach using IQTREE2 v.2.1.3 [29]. We selected the best partitioning scheme and substitution models with ModelFinder [30] using the option implemented in IQ-TREE2 (-m MFP+MERGE). For the tree reconstruction, 1000 ultrafast bootstrapping (UFBoot) was used to evaluate node support (-B 1000) [31,32]. To reduce the risk of overestimating branch support in the UFBoot test, we implemented the -bnni option with 10 rounds of IQTREE (--runs 10) to avoid suboptimal typologies. We considered as supported nodes those with bootstrap (BS) values above 95. To further support our results, we also carried out phylogenetic analyses with a Bayesian approach, using MrBayes 3.2.7 implemented in the Cipress Portal v3.1. We used the GTR+I+G as a substitution model for all matrices, 5,000,000 mcmc with an initial burn-in of 10%, and a subsampling every 1000 chains. We considered as supported nodes those with posterior probability (PP) values above 0.9. We visualized and edited the resulting trees in ITOL v.6.8 [33].

### 2.4. Species Delimitation

We employed three species delimitation methods for delineating MOTUs with the *cox1* and *cox2* matrix. First, we used the Assemble Species by Automatic Partitioning (ASAP), which bases species partitions on single locus sequence alignments and their pairwise genetic distance [34]. The ASAP web server is available at https://bioinfo.mnhn.fr/abi/public/asap/, accessed on 30 September 2024. We carried out the Jukes-Cantor (JC69), Kimura (K80), and p-distances methods using default settings for our analysis, and we kept the best ASAP score for each of them. Second, we used PTP approaches, which base their species partitions on phylogenetic topologies, using the bPTP web server (https://species.h-its.org/, accessed on 30 September 2024). We evaluated the species partitions with mPTP (multi-rate Poisson Tree Processes) [35] and bPTP (Bayesian Poisson Tree Process) [36], both of them having as input files the *cox1* and *cox2* ML trees, previously inferred with IQTREE2, and cropping the outgroup *Seira* to avoid aberrant results due to its external, distant position. We illustrated the result of the lowest ASAP scores and the result provided by the PTP approaches next to the Maximum Likelihood trees.

### 2.5. Morphological Analyses

**Figure 2:**
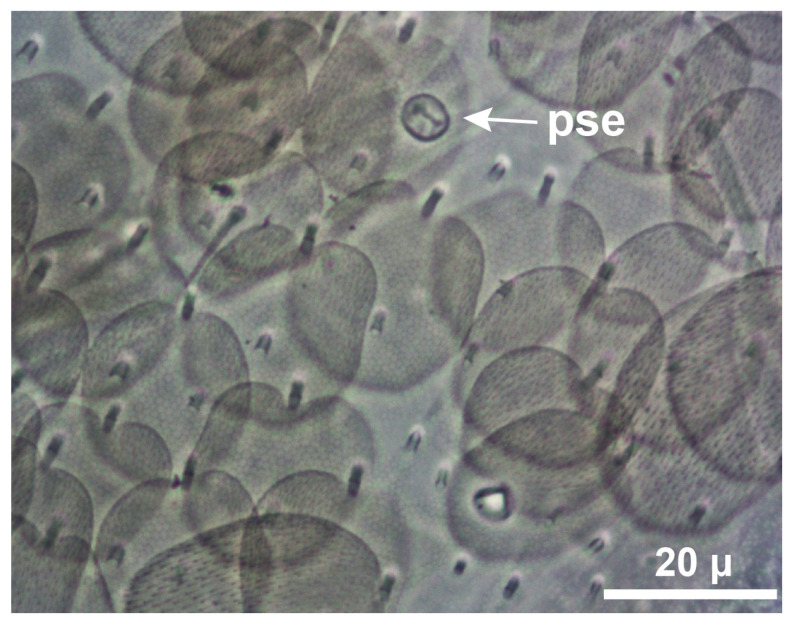
Pseudopore. Body region of a *Lepidocyrtus* specimen showing a pseudopore (pse).

For morphological approaches, the specimens were mounted on slides with the head separated from the body. The specimens were cleared using Nesbitt’s fluid and then slide-mounted in Hoyer medium. The slides were studied under a phase contrast microscope (Axio Scope A1, manufactured by Zeiss, Oberkochen, Germany) with a digital camera attached (CMEX Pro 5, manufactured by Euromex Microscopen, Duiven, The Netherlands). Morphological and chaetotaxic characters of the dorsal, lateral, and ventral regions of the head, body, and appendages were analyzed. A detailed description of the quantity and arrangement of pseudopores was also made (Figure 2).

**Figure A1:**
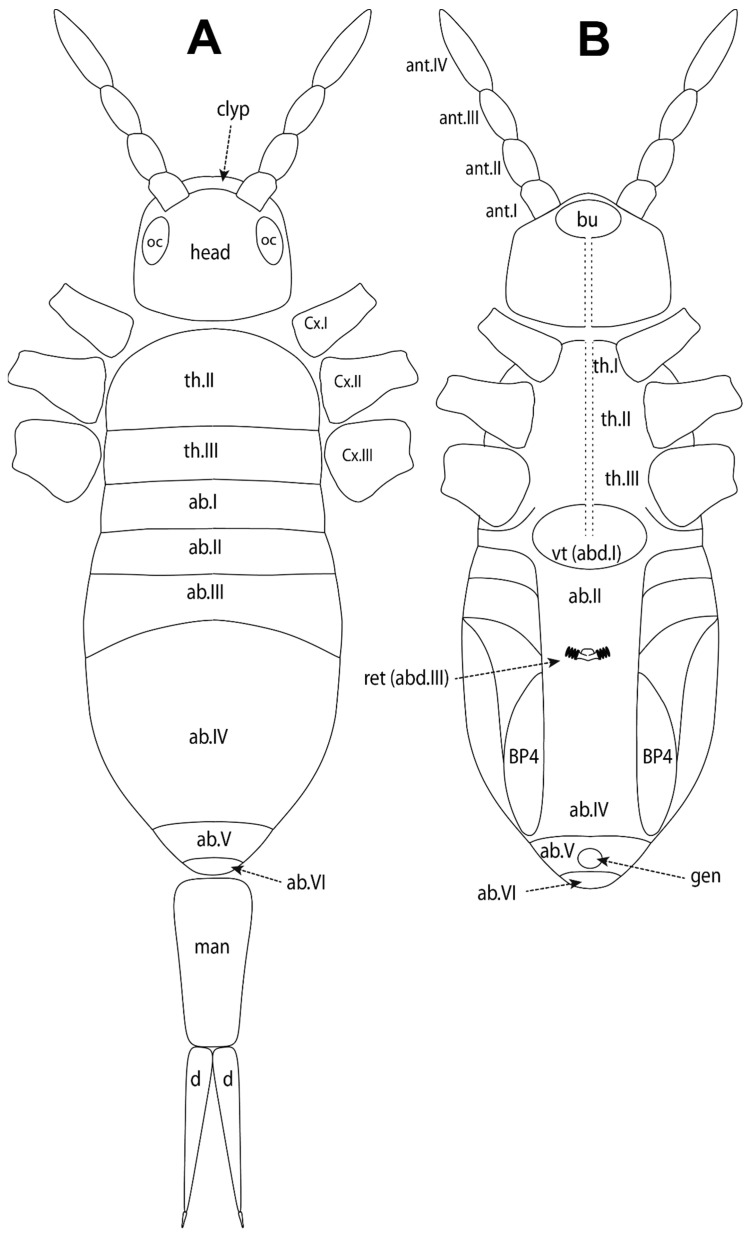
Body segments from where the presence and abundance of pseudopores have been evaluated. (**A**) Dorsal scheme of pseudopores areas. (**B**) Ventral and lateral scheme of pseudopore areas.

The following abbreviations have been used in tables, figures, and morphological descriptions: ab., abdominal segment; ant., antennal segment; BP4, basal plate of the fourth abdominal segment; bu, buccal area; clyp, clypeus; cx, coxa; d, dentes; gen, genital plate; man, manubrium; oc, ocular area; pse, pseudopore; ret, retinaculum; th, thoracic segment; vt, ventral tube; and I–VI, segment (Figure A1). For collections, we used the abbreviation MIUP (Museo de Invertebrados de la Universidad de Panamá, Ciudad de Panamá, Panama).

## 3. Results

### 3.1. Phylogenetic Approximations

A total of 72 specimens were analyzed molecularly (Table 1 and Table 2). The *cox1* matrix consisted of 40 specimens of *Lepidocyrtus* from Panama, Brazil, Costa Rica, and USA, totaling 658 base pairs. The *cox2* matrix included 38 specimens of *Lepidocyrtus* and 684 base pairs. Finally, the *EF-1α* matrix consisted of 36 specimens of *Lepidocyrtus* and 851 base pairs. All the matrices included three *Seira* specimens as outgroups.

Both maximum likelihood and Bayesian inference provided the same topology on the mitochondrial *cox1*, *cox2*, and concatenated matrices. The nuclear *EF-1α* provided different topologies between phylogenetic inference methods. Overall, the Bayesian inference topologies were better supported.

**Figure 3:**
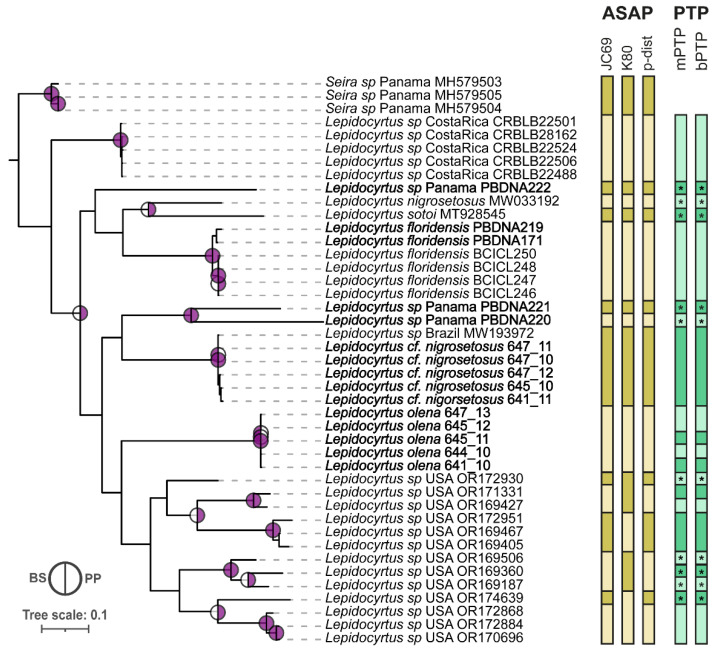
*Lepidocyrtus* phylogenetic tree and species delimitation using the molecular marker *cox1***.** Only supported nodes are represented with circles in the phylogeny. The colored left half of the circles corresponds to BS support obtained with ML approaches, and the colored right half of the circles corresponds to PP support obtained with Bayesian approaches. Individuals with newly generated sequences are highlighted in bold font. Distance-based (ASAP) and topological-based (PTP) indicators are displayed in the right panel. Supported species delimitations are displayed for mPTP and bPTP with asterisks.

The *cox1* matrix showed *Lepidocyrtus* sp. from Costa Rica as the first splitting lineage within the genus, with no support (Figure 3). Following, there is a split between a clade composed of *Lepidocyrtus* sp. Panama PBDNA222, *L. nigrosetosus*, *L. sotoi*, *Lepidocyrtus* sp. Panama PBDNA219 and PBDNA171, and *L. floridensis* from all the other specimens (BS < 95; PP > 0.9). Within this clade, the node of *L. nigrosetosus* and *L. sotoi* was supported by PP but not BS, as it also supported the node separating *Lepidocyrtus* sp. PBDNA 219 and 171 from *L. floridensis* (BS > 95; PP > 0.9) (Figure 3). The next split in the phylogeny (BS < 95; PP < 0.9) generated a clade that involved two siblings *Lepidocyrtus* sp. from Panama (PBDNA220 and PBDNA221) (BS > 95; PP > 90) and a clade composed of five *Lepidocyrtus* sp. from Panama and *Lepidocyrtus* sp Brazil MW193972 (Figure 3). Following, five Panamanian *Lepidocyrtus* were separated from all USA *Lepidocyrtus*, yet without support. Within the USA clade, our results identified two non-supported main clades (Figure 3).

**Figure A2:**
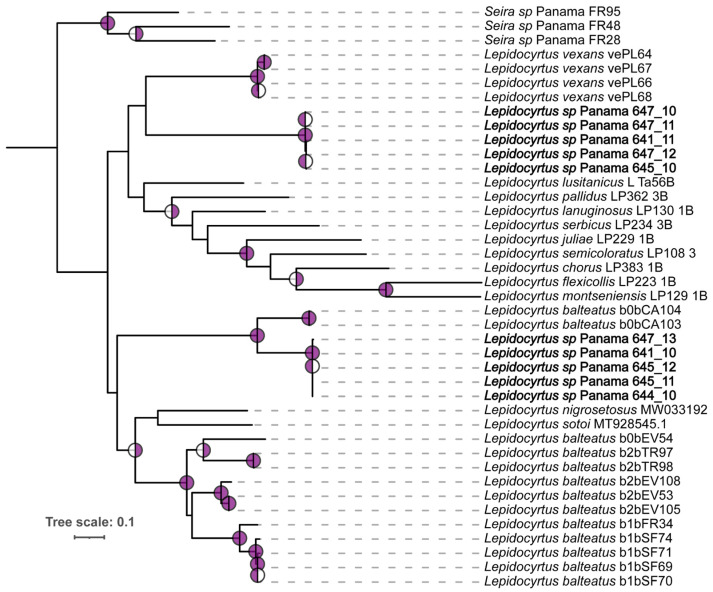
*Lepidocyrtus* phylogenetic tree using the molecular marker *cox2*. Only supported nodes are represented with circles in the phylogeny. The left half of the circles corresponds to BS support obtained with ML approaches, and the right side corresponds to PP support obtained with Bayesian approaches. Individuals with newly generated sequences are highlighted in bold font.

The *cox2* matrix placed five *Lepidocyrtus* sp. individuals from Panama as the sibling clade of *L. vexans*, yet without support (Figure A2). The remaining five Panamanian *Lepidocyrtus* sp. individuals were found to be the sibling clade to a lineage composed of two *L. balteatus*, supported with both BS and PP (Figure A2). The *cox2* topology was coincident with the one obtained with the concatenated matrix.

**Figure A3:**
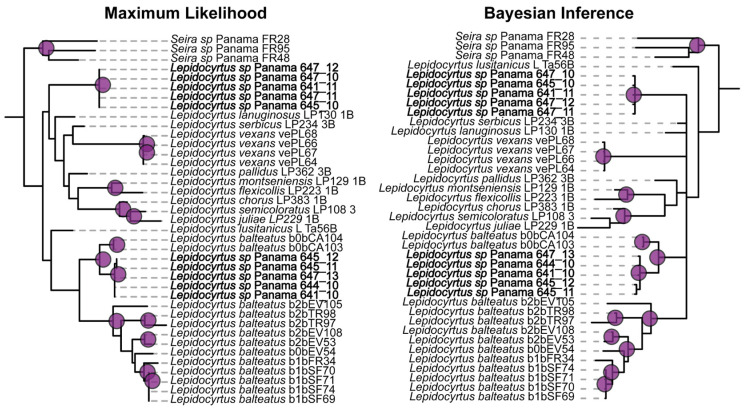
*Lepidocyrtus* phylogenetic tree using the molecular marker *EF-1α*. Only supported nodes are represented with circles in the phylogeny. The phylogenetic tree on the left was obtained using maximum likelihood approaches. The phylogenetic tree on the right was obtained using Bayesian inference approaches. Individuals with newly generated sequences are highlighted in bold font.

The nuclear *EF-1α* provided two topologies depending on the phylogenetic inference approach (Figure A3). Under a maximum likelihood framework, five *Lepidocyrtus* sp. from Panama were siblings to a clade composed of *L. vexans* and all European species but *L. lusitanus*, without node support (Figure A2). The other five Panamanian *Lepidocyrtus* sp. were siblings to a clade composed of two individuals of *L. balteatus*, supported by both BS and PP (Figure A3). Conversely, the Bayesian inference approach resulted in a large polytomy hosting seven lineages (Figure A3). Five *Lepidocyrtus* sp. individuals split on the polytomy, and the other five *Lepidocyrtus* sp. specimens were siblings to the clade composed of two *L. balteatus*, supported with BS and PP (Figure A3).

**Figure 4:**
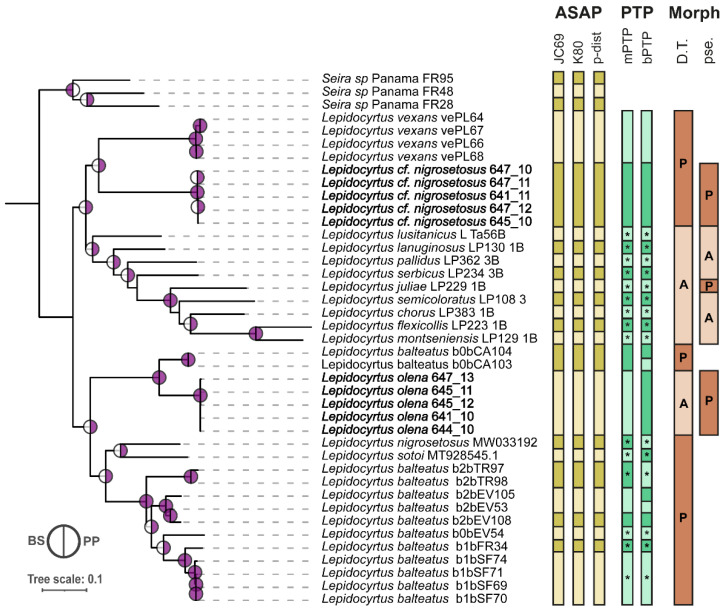
*Lepidocyrtus* phylogenetic tree using the concatenated matrix using the molecular markers *cox2* and *EF-1α*, and species delimitation based on *cox2*. Only supported nodes are represented with circles in the phylogeny. The colored left half of the circles corresponds to BS support obtained with ML approaches, and the colored right half of the circles corresponds to PP support obtained with Bayesian approaches. Individuals with newly generated sequences are highlighted in bold font. Distance-based (ASAP), topological-based (PTP), and morphological-based delimitations are displayed in the right panel. Supported species delimitations are displayed for mPTP and bPTP with asterisks. For morphology, D.T. stands for dental tubercle, and pse stands for pseudopores on BP4 region. The presence of the morphological traits is represented with a P inside of the boxes, and their absence with an A.

The concatenated matrix was, in comparison to gene tree topologies, much better supported. The resulting topology (Figure 4) showed two main clades, although without support. The first clade involved all the European species, *L. vexans*, and five Panamanian *Lepidocyrtus* sp. The clade of *Lepidocyrtus* sp. from Panama was the sibling of *L. vexans* supported with PP (BS < 95). The clade involving these two species was the sibling to a clade composed of all European *Lepidocyrtus*, supported with PP (BS<95). The other main clade showed a first split (BS > 95; PP > 0.9), separating a clade involving Panamanian *Lepidocyrtus* sp. as a sibling to two *L. balteatus* (BS < 95; PP > 0.9), and these species siblings to the remaining species labeled as *L. balteatus, L. nigrosetosus,* and *L. sotoi*. For the latter clade, *L. nigrosetosus* was the sibling of *L. sotoi* (BS <9 5; PP > 0.9), and this clade was the sibling of the remaining *L. balteatus* specimens (BS < 95; PP > 0.9).

### 3.2. Species Delimitation Analyses

The *cox1* ASAP analysis, using the JC69, K2P, and p-distance approaches, identified 16, 14, and 16 groups, respectively (Figure 3). All approaches produced consistent partitioning for the specimens from Panama, Costa Rica, and Brazil, and minor differences were observed in the partitioning of *Lepidocyrtus* specimens from the USA. Specifically, the ASAP analyses using *cox1* sequences grouped all *Lepidocyrtus* sp. sequences from Costa Rica into one cluster, *L. sotoi* into another, and *L. nigrosetosus* into a separate group. Additionally, four sequences of *L. floridensis* from Panama, along with two newly collected samples (PBDNA171-PBDNA219), formed another group. The specimen identified as *Lepidocyrtus* sp. *Brazil* MW193972 from Brazil was grouped with five *Lepidocyrtus* sp. from Panama, while the remaining seven *Lepidocyrtus* from Panama were divided into three distinct groups: *Lepidocyrtus* sp. PBDNA220, *Lepidocyrtus* sp. PBDNA221, and *L. olena* (five specimens).

Using the *cox1* dataset, the mPTP and bPTP analyses delimited twenty-two putative species, with some groups being over-split, though only a few received strong support. In the analyses, all *Lepidocyrtus* sp. from Costa Rica were clustered together without support, while *L. sotoi* and *L. nigrosetosus* formed distinct groups, both with high support. Additionally, four sequences of *L. floridensis* from Panama, along with two newly collected *Lepidocyrtus* sp. specimens (PBDNA171 and PBDNA219), formed a separate cluster. The specimen *Lepidocyrtus* sp. Brazil MW193972 (from Brazil) was grouped with five *Lepidocyrtus* sp. from Panama, whereas seven newly sequenced *Lepidocyrtus* specimens from Panama were split into six distinct groups, two of which had high support.

The *cox2* matrix delimitation analyses with ASAP provided consistent results regardless of the JC69, K80, or p-distance approximation (Figure 4). All *Lepidocyrtus* from NCBI previously labeled as distinct species were indeed recovered as independent species, except *L. balteatus*, which was split into seven different species. The clades composed with the Panamanian specimens *Lepidocyrtus* sp. 647_10, 647_11, 641_11, 647_12, and 645_10 (identified as *Lepidocyrtus* spusing *cox1*) and the clade including *Lepidocyrtus* sp. 647_13, 645_11, 645_12, 641_10, and 644_10 were identified as two separate species. All *Seira* specimens were recovered as distinct putative species. The mPTP analyses were in agreement with the results provided by ASAP. Nevertheless, the bPTP analysis further split *L. balteatus* into nine putative species. All European species delimitations were supported by both methods, as well as the delimitation of *L. nigrosetosus*, *L. sotoi*, and four *L. balteatus* lineages.

### 3.3. Taxonomic Results

#### 3.3.1. *Lepidocyrtus floridensis* Snider, 1967

Studied material for molecular analyses: Sample PBVOU068, 1 juvenile (PBDNA219); and sample PBVOU184, 1 juvenile (PBDNA171).

Distribution: Florida (type locality), Cuba [19], Mexico [37,38], and Panamá [23].

Diagnosis: No adult specimens were obtained in this study, and therefore they have not been studied morphologically. The sequences obtained have been compared with the sequences of Panamanian specimens from Basset et al. (2022) [23], who also used the *cox1*. The similarity of our sequences with the *cox1* sequences of *L. floridensis* in their work has allowed us to assign our specimens to this species.

#### 3.3.2. *Lepidocyrtus* cf. *nigrosetosus* Folsom, 1927

Figure 5, Table 3

**Figure 5:**
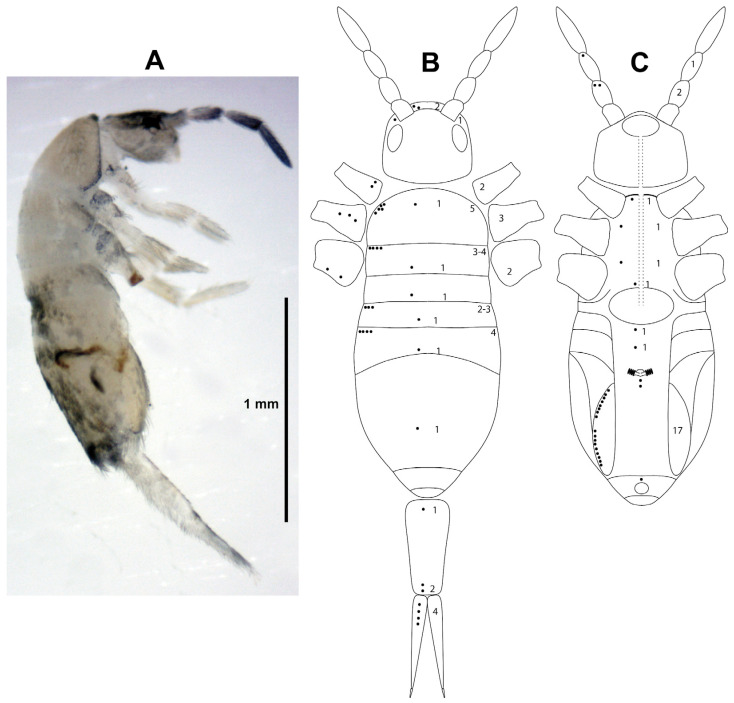
*Lepidocyrtus* cf. *nigrosetosus*. (**A**) Lateral habitus. (**B**) Dorsolateral scheme of pseudopores distribution. (**C**) Ventrolateral and lateral scheme of pseudopores distribution. In figures (**B**,**C**), the arrangement of the pseudopores (dots) is indicated on the left, and the range of pseudopores that may be present on the right.

Studied material for molecular and morphology analyses: Sample PBVOU120, 1 adult (LP641-11; MIUP); sample PBVOU095, 1 adult (LP645-10; MIUP); and sample PBVOU184, 3 adults (LP647-10 to LP647-12; MIUP).

Studied material for morphology analyses: Sample PBVOU057, 1 adult ♀ (LP640-1; MIUP); sample PBVOU120, 1 juvenile (LP641-2; MIUP) and 1 juvenile (LP641-3; MIUP); sample PBVOU048, 1 juvenile (LP642-1; MIUP); sample PBVOU065, 1 juvenile (LP643-1; MIUP); sample PBVOU068, 1 juvenile (LP644-1; MIUP); sample PBVOU049, 1 juvenile (LP646-1, MIUP); and sample PBVOU184, 1 juvenile (LP647-5; MIUP), 1 juvenile (LP647-6; MIUP), 1 adult ♀ (LP647-7; MIUP), and 1 juvenile (LP647-8; MIUP).

Distribution: Puerto Rico (type locality), Colombia, Jamaica [19], Galápagos [39], US Virgin Islands [9,40], Brazil [41], Nevis [42], and Panamá (present paper).

Diagnosis: Adult specimens with a body length of 1.6 mm (without head) (Figure 5A). The 11 examined specimens present all the characters detailed for this species in the original description by Folsom (1927) [22], as well as in the additional characters included in this species’ redescriptions of Mari Mutt (1986) [43] and Katz et al. (2016) [39]. The complete distribution of pseudopores for this species has not been described so far. Table 3 and Figure 5B,C indicate the position and quantity of these integumentary structures on the head, body, and appendages. The presence and absence of the dental tubercle and the pseudopores are represented within a phylogenetic framework in Figure 4.

Remarks: Although our specimens present all the characteristics of *L. nigrosetosus*, we have chosen to name them *L.* cf. *nigrosetosus*. This is due to two reasons: First, the genetic sequences of our specimens and the published sequences of the species *L. nigrosetosus* are very far apart in the phylogenies we have carried out. Second, several Neotropical species are very similar to *L. nigrosetosus*, all of them included in the so-called “*Lepidocyrtus nigrosetosus* species group” by Bernard et al. (2015) [44] and Cipola et al. (2019) [45], and the descriptions of some of them are not exhaustive enough to make a precise identification. The descriptions of *Lepidocyrtus geayi* Denis, 1924 (French Guiana), and *Lepidocyrtus schmidti* Handschin, 1927 (Costa Rica), are quite incomplete, and, as Mari-Mutt (1986) stated [43], fresh material must be obtained before a detailed comparison can be made. Our *L.* cf. *nigrosetosus* are also very similar to *Lepidocyrtus amazonicus* Cipola and Bellini, 2019 (Brazilian Amazon). Both species present pseudopores in the laterodorsal region of thoracic segments II and III and some abdominal segments [45]. Unfortunately, the description of the Brazilian species does not state the presence or absence of pseudopores in BP4.

*Lepidocyrtus nigrosetosus* is a generalist species capable of tolerating high temperatures and low humidities. This species has a wide distribution throughout the Caribbean regions [19,43], and its presence in Puerto Rico is possibly due to a historical human-mediated introduction [46]. It commonly inhabits leaf litter in disturbed habitats, including urban forest patches and marginal wooded areas [42].

#### 3.3.3. *Lepidocyrtus olena* Christiansen and Bellinger, 1992

Figure 6, Table 3

**Figure 6:**
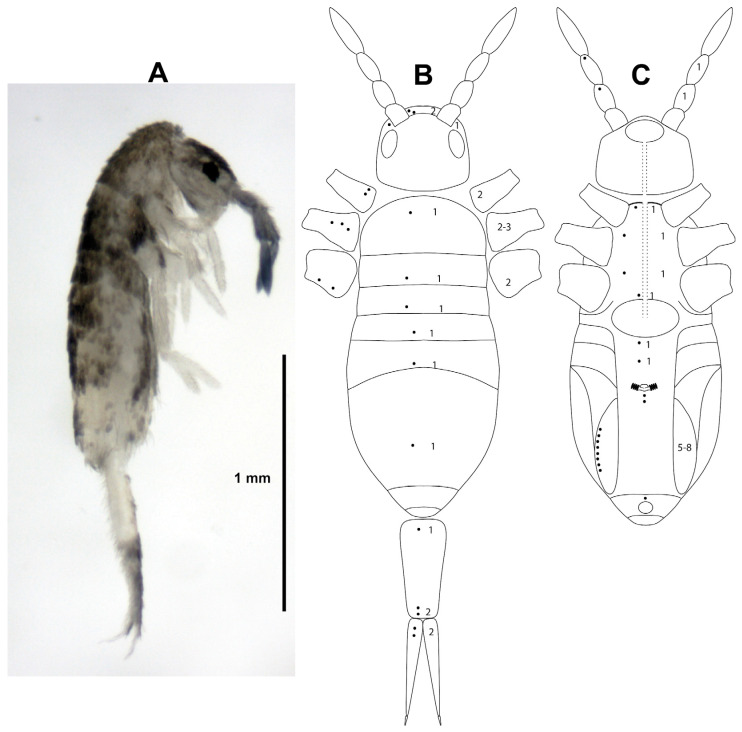
*Lepidocyrtus olena*. (**A**) Lateral habitus. (**B**) Dorsolateral scheme of pseudopores distribution. (**C**) Ventrolateral and lateral scheme of pseudopores distribution. In figures (**B**,**C**), the arrangement of the pseudopores (dots) is indicated on the left, and the range of pseudopores that may be present on the right.

Studied material for molecular and morphology analyses: Sample PBVOU120, 1 adult (641-10; MIUP); sample PBVOU068, 1 adult (LP644-10; MIUP); sample PBVOU095, 2 adults (LP645-11, LP645-12; MIUP); and sample PBVOU184, 1 adult (LP647-13; MIUP).

Studied material for morphology analyses: - Sample PBVOU120, 1 juvenile (LP641-1; MIUP); sample PBVOU095, 2 juveniles (LP645-1, LP645-2; MIUP); and sample PBVOU184, 1 juvenile (LP647-9; MIUP).

Distribution: Hawaii (type locality [47]), Rapa Nui [44], and Panamá (present paper). This is the first time this species has been cited in Panama and the Neotropical region.

Diagnosis: None of the four specimens morphologically examined had a developed genital plate. The body length (without head) was 0.4–1.2 mm (Figure 6A). The specimens presented all the characters indicated for this species in the original description by Christiansen and Bellinger (1992) [47] and the redescription of Bernard et al. (2015) [44]. The complete distribution of pseudopores for this species has not been described so far. Table 3 and Figure 6B,C indicate the position and quantity of these integumentary structures on the head, body, and appendages. The presence and absence of the dental tubercle and the pseudopores are represented within a phylogenetic framework in Figure 4.

Remarks: The presence on Rapa Nui and Panama of a species originally described from the Hawaiian Islands may be due to human activities, ancient or modern, across the Pacific Ocean. As indicated by Bernard et al. (2015) [44], it is not clear that *L. olena* is native to Hawaii. Its presence in Panama and its molecular and morphological similarity with species from the Caribbean support the theory that it is a native species of the Neotropical region and may have colonized the Pacific Islands within shipments of ornamental or agricultural plants from the South American continent.

#### 3.3.4. *Lepidocyrtus* sp1, sp2 and sp3

Studied material for molecular analyses: Sample PBVOU058, 1 juvenile (PBDNA222–sp1); sample PBVOU158, 1 juvenile (PBDNA221–sp2); and sample PBVOU116, 1 juvenile (PBDNA220–sp3).

Diagnosis: The three specimens obtained from these species have not been studied morphologically but only molecularly, as they were too small.

## 4. Discussion

This study highlights the remarkable diversity of *Lepidocyrtus* species in Panama’s Parque Natural Metropolitano (PNM), where six distinct species were identified, three potentially new to science and two others recorded in Panama for the first time. Our results also reveal that the morphological characters, such as the dental tubercle and pseudopores, exhibit considerable homoplasy, evolving independently across different lineages (Figure 4). While these characters assist in distinguishing species, they lack the consistency needed for higher taxonomic classifications. By integrating molecular analyses with morphological traits, the research emphasizes the importance of molecular data for accurate species delimitation and reinforces PNM’s role as a conservation priority in the Neotropics, advocating for comprehensive approaches to preserve soil diversity.

### 4.1. Lepidocyrtus nigrosetosus Species Group

Bernard et al. (2015) [44] considers the “*Lepidocyrtus nigrosetosus* species group”, a group of species sharing the following characters: blunt, anteriorly produced mesothoracic hood, no head macrochaetae between A0 and Pa5, anterior row of post-labial chaetae smooth, abd IV with four median macrochaetae (Sm, B4–6), and truncate prothoracic unguiculus. In this group, Bernard et al. (2015) includes the species *L. geayi* Denis, 1924 (French Guiana), *L. geayides* Denis, 1931 (Costa Rica), *L. nigrosetosus* (Puerto rico), *L. olena* (Hawaii), and *L. schmidti* Handschin, 1927 (Costa Rica). Later, Cipola et al. 2019 [45] includes in the group the species *L. amazonicus* (Brazil) and *L. sotoi* (Brazil).

Our molecular results do not support the monophyly of the “*Lepidocyrtus nigrosetosus* species group” as defined by Bernard et al. (2015) [44], since the studied specimens of *L. olena* and *L.* cf. *nigrosetosus* are inserted in two well-differentiated clades in the phylogenies.

*Lepidocyrtus olena* presents morphological characters that clearly differentiate it from the rest of the species that are included in the *L. nigrosetosus* species group: absence of scales in the dorsal region of ant.I, short and ciliated labial chaeta R, presence of cephalic macrochaeta pa5, presence of chaetae a2p and me3 in the dorsal region of abd.II, and absence of dental tubercle.

Godeiro et al. (2021) [48] published mitogenome sequences of a specimen of *L. nigrosetosus* from Brazil, from which we have extracted the MW033192 sequences used in the phylogenetic analyses presented in this study. The *cox1*, *cox2*, and *EF-1α* sequences of our specimens, identified as *L.* cf. *nigrosetosus,* differ markedly from those of the specimen studied by Godeiro et al. (2021) [48]. This discrepancy suggests that the morphological characters currently used for the diagnosis of this species do not allow an unequivocal identification. So, we are presumably facing a case of uncovered morphospecies within *L. nigrosetosus*. Our findings support that morphological characters alone offer lower taxonomic resolution compared to the haplotypic diversity revealed by molecular data, underscoring the need for additional diagnostic markers.

In the *cox1* tree, the sequence MW193972 corresponds to a *Lepidocyrtus* specimen from Mato Grosso, Brazil (F.G. Oliveira pers.com, 1 October 2024) and matches 100% with the sequences of our *L.* cf. *nigrosetosus* specimens from Panama, indicating that the species we found in Panama may have a wide distribution along the Neotropical area. The existence of species complexes in the genus *Lepidocyrtus* has been detected in several studies using molecular data [4,6,7,8,9,25]. The *L. nigrosetosus* group is defined solely by morphological data, and the molecular data analyzed in this work shed light on the true nature of this group. Thus, in the phylogenies carried out, the reference clade for the *L. nigrosetosus* group is formed by the specimens *L. nigrosetosus* MW033192 and *L. sotoi* MT928545. In none of these phylogenies are the Panamanian *L. cf nigrosetosus* joined to this reference clade, which indicates that the *L. nigrosetosus* group is not monoyphyletic.

### 4.2. Dental Tubercle and Lepidocyrtus Subgenera

Based on species from the Oriental or Palearctic regions, the genus *Lepidocyrtus* was conveniently divided into several subgenera [49,50]. These authors used the presence or absence of scales on antennae, legs, collophore and dorsal manubrium, and the shape of dental tubercle as basic diagnostic characters. However, the proposed subgenera and the characters used to describe them are controversial and have largely raised debate among taxonomists [45,51].

For Neotropical species, the presence of scales on appendages does not seem to be a valid character for the diagnosis of the proposed subgenera [9,46,52]. Likewise, the studies in which the presence and shape of the dental tubercle are analyzed do not seem to unequivocally support its validity as a subgeneric diagnostic character. Christiansen and Bellinger (1991) [52], analyzing phylogenetic relationships among Hawaiian species of *Lepidocyrtus*, concluded that the dental tubercle was not important in defining monophyletic lineages in the region. The phylogenetic study of Soto-Adames (2000) analyzing Neotropical (mainly Puerto Rican) *Lepidocyrtus* species partially supports the suitability of the dental tubercle as a subgeneric diagnostic character and that it is only restricted to the Neotropical species [46]. To date, *Lepidocyrtus thomosvaryi* Winkler and Traser, 2012, is the only European species with dental tubercle, and molecular phylogenies clearly place this species within a species-group formed by species without dental tubercle [53], indicating the unsuitability of this character to define supraspecific taxa in the European fauna.

In our phylogenies, the character dental tubercle showed a very homoplastic behavior, evolving independently on each clade, biogeographic region, and species complex. Thus, although we consider it has an evident relevance for species diagnosis, it does not seem suitable for diagnostic purposes at higher taxonomic levels.

### 4.3. Usage of Pseudopores and New Taxonomic Trait

We have described for the first time the abundance and distribution of the pseudopores in *L.* cf. *nigrosetosus* and *L. olena*. Focusing on the BP4 region, we have combined this information with the previous knowledge of European species. So far, there are four described European species with pseudopores on BP4: *L. juliae* Mateos, 2011 (from Crete, Greece), *L. milagrosae* Mateos and Álvarez-Presas, 2021 (from Rhodes, Greece), and *L. cf lignorum* (population from Moldova) and *L.* cf. *violaceus* (population from Paros, Greece) [5]. The first of these species has been included in our *cox2* and *EF-1α* phylogenetic analyses.

In the Neotropics, the only published information on the distribution of pseudopores in *Lepidocyrtus* species is that of Cipola and Viana (2023) [14] in reference to the subgenus *Fractocyrtus*. All four species comprising this subgenus have pseudopores in the BP4 region, but none of them have been included in our analysis. There is no published information on the presence or absence of pseudopores in the BP4 region for the species *L. vexans*, *L. nigrosetosus*, *L. sotoi,* or *L. balteatus*, thus hampering our comparisons and its evaluation as a phylogenetic trait of some groups.

We found that the two Neotropical species *L.* cf. *nigrosetosus* and *L. olena* and the European species *L. juliae*, despite not being monophyletic, present pseudopores in BP4. This indicates that this character is not appropriate for defining supraspecific entities. In any case, the presence of pseudopores in BP4 is a useful character for the diagnosis of *Lepidocyrtus* species, which, in combination with the dental tubercle, could help to describe the species and quickly assess regional biodiversity at low cost.

## 5. Conclusions

Our study has demonstrated that the Parque Natural Metropolitano, and by extension, the Mesoamerican region, is a hotspot of *Lepidocyrtus* species. By installing two sampling plots separated 200 m one from the other, with a total of eight pitfall traps, we have recovered six species. Interestingly, two of the species represent the first record for Panama or the whole American continent, and three of them are postulated as potentially new species based on molecular data. This diversity was achieved by studying only 30 specimens, demonstrating that the ecosystem is well-balanced and there is no dominance of a few species over the others. Moreover, the genetic richness of only 0.1 ha of the PNM overtakes the Panamanian diversity described in Cicconardi et al. 2013 [8], as we have recovered species across the whole *Lepidocyrtus* Tree of Life. Our study strengthens the importance of addressing Panamanian *Lepidocyrtus* molecularly and morphologically, since many species may still be unknown to science, as well as remarks on the importance of the Parque Natural Metropolitano for biological welfare.

## Figures and Tables

**Table 1:** Information on the *Lepidocyrtus* samples collected in Panama used in the present study. For each sample, we provide its ID based on DNA barcoding, country, voucher code, specimen code, DNA extraction code, and GenBank accession numbers for *cox1*, *cox2*, and *EF-1α* genes.

Species ID (DNA-based)	Sample Code	Specimen Code	DNA Code	*cox1*	*cox2*	*EF-1α*
*Lepidocyrtus* cf. *nigrosetosus*	PBVOU048	LP642-1	NA	NA	NA	NA
*Lepidocyrtus* cf. *nigrosetosus*	PBVOU049	LP646-1	NA	NA	NA	NA
*Lepidocyrtus* cf. *nigrosetosus*	PBVOU065	LP643-1	NA	NA	NA	NA
*Lepidocyrtus* cf. *nigrosetosus*	PBVOU068	LP644-1	NA	NA	NA	NA
*Lepidocyrtus* cf. *nigrosetosus*	PBVOU095	LP645_10	LP645_10	PQ539764	PQ536686	PQ536690
*Lepidocyrtus* cf. *nigrosetosus*	PBVOU120	LP641_11	LP641_11	PQ539762	PQ536687	PQ536692
*Lepidocyrtus* cf. *nigrosetosus*	PBVOU120	LP641-2	NA	NA	NA	NA
*Lepidocyrtus* cf. *nigrosetosus*	PBVOU120	LP641-3	NA	NA	NA	NA
*Lepidocyrtus* cf. *nigrosetosus*	PBVOU184	LP647_10	LP647_10	PQ539768	PQ536684	PQ536691
*Lepidocyrtus* cf. *nigrosetosus*	PBVOU184	LP647_11	LP647_11	PQ539769	PQ536683	PQ536689
*Lepidocyrtus* cf. *nigrosetosus*	PBVOU184	LP647_12	LP647_12	PQ539770	PQ536685	PQ536688
*Lepidocyrtus* cf. *nigrosetosus*	PBVOU184	LP647-5	NA	NA	NA	NA
*Lepidocyrtus* cf. *nigrosetosus*	PBVOU184	LP647-6	NA	NA	NA	NA
*Lepidocyrtus* cf. *nigrosetosus*	PBVOU184	LP647-7	NA	NA	NA	NA
*Lepidocyrtus* cf. *nigrosetosus*	PBVOU184	LP647-8	NA	NA	NA	NA
*Lepidocyrtus floridensis*	PBVOU068	PBDNA219	PBDNA219	PQ539772	NA	NA
*Lepidocyrtus floridensis*	PBVOU184	PBDNA171	PBDNA171	PQ539771	NA	NA
*Lepidocyrtus olena*	PBVOU068	LP644_10	LP644_10	PQ539763	PQ536681	PQ536694
*Lepidocyrtus olena*	PBVOU095	LP645_11	LP645_11	PQ539765	PQ536680	PQ536697
*Lepidocyrtus olena*	PBVOU095	LP645_12	LP645_12	PQ539766	PQ536679	PQ536696
*Lepidocyrtus olena*	PBVOU095	LP645-1	NA	NA	NA	NA
*Lepidocyrtus olena*	PBVOU095	LP645-2	NA	NA	NA	NA
*Lepidocyrtus olena*	PBVOU120	LP641_10	LP641_10	PQ539761	PQ536682	PQ536695
*Lepidocyrtus olena*	PBVOU120	LP641-1	NA	NA	NA	NA
*Lepidocyrtus olena*	PBVOU184	LP647_13	LP647_13	PQ539767	PQ536678	PQ536693
*Lepidocyrtus olena*	PBVOU184	LP647-9	NA	NA	NA	NA
*Lepidocyrtus* sp.	PBVOU058	PBDNA222	PBDNA222	PQ539774	NA	NA
*Lepidocyrtus* sp.	PBVOU116	PBDNA220	PBDNA220	PQ539773	NA	NA
*Lepidocyrtus* sp.	PBVOU158	PBDNA221	PBDNA221	PQ539775	NA	NA

**Table 2:** Information on the samples downloaded from the public repositories BOLD and GenBank. For each sample, we provide the species, country where it was collected, specimen code, and accession numbers for *cox1*, *cox2*, and *EF-1α.* Accession numbers in bold font indicate they were retrieved from BOLD, whereas accession numbers in regular font indicate they were retrieved from GenBank. Codes with an asterisk indicate these were retrieved from full mitochondrial genome assemblies.

Species	Country	Code	*cox1*	*cox2*	*EF-1α*
*Lepidocyrtus balteatus*	Panama	b1bFR34	NA	KF364880	KF364773
*Lepidocyrtus balteatus*	Panama	b2bEV53	NA	KF364894	KF364792
*Lepidocyrtus balteatus*	Panama	b0bEV54	NA	KF364895	KF364794
*Lepidocyrtus balteatus*	Panama	b1bSF69	NA	KF364909	KF364811
*Lepidocyrtus balteatus*	Panama	b1bSF70	NA	KF364910	KF364812
*Lepidocyrtus balteatus*	Panama	b1bSF71	NA	KF364911	KF364813
*Lepidocyrtus balteatus*	Panama	b1bSF74	NA	KF364914	KF364817
*Lepidocyrtus balteatus*	Panama	b2bTR97	NA	KF364932	KF364836
*Lepidocyrtus balteatus*	Panama	b2bTR98	NA	KF364933	KF364838
*Lepidocyrtus balteatus*	Panama	b0bCA103	NA	KF364938	KF364841
*Lepidocyrtus balteatus*	Panama	b0bCA104	NA	KF364939	KF364842
*Lepidocyrtus balteatus*	Panama	b2bEV105	NA	KF364940	KF364843
*Lepidocyrtus balteatus*	Panama	b2bEV108	NA	KF364943	KF364844
*Lepidocyrtus chorus*	Croatia	LP383	NA	MF095522	MF095609
*Lepidocyrtus flexicollis*	Spain	LP223	NA	MF095485	MF095587
*Lepidocyrtus floridensis*	Panama	BCICL246-19	**BCICL246-19**	NA	NA
*Lepidocyrtus floridensis*	Panama	BCICL247-19	**BCICL247-19**	NA	NA
*Lepidocyrtus floridensis*	Panama	BCICL248-19	**BCICL248-19**	NA	NA
*Lepidocyrtus floridensis*	Panama	BCICL250-19	**BCICL250-19**	NA	NA
*Lepidocyrtus juliae*	Greece	LP229	NA	MF095490	MF095590
*Lepidocyrtus lanuginosus*	Spain	LP130	NA	MF095474	MF095579
*Lepidocyrtus lusitanicus*	Spain	L Ta56B	NA	MF095537	MF095614
*Lepidocyrtus montseniensis*	Spain	LP129	NA	MF095470	MF095571
*Lepidocyrtus nigrosetosus*	Brazil	MW033192	MW033192 *	MW033192 *	NA
*Lepidocyrtus pallidus*	Norway	LP362	NA	MF095519	MF095608
*Lepidocyrtus semicoloratus*	Greece	LP108	NA	MT345919	MT345985
*Lepidocyrtus serbicus*	Greece	LP234	NA	MF095495	MF095591
*Lepidocyrtus sotoi*	Brazil	MT928545	MT928545 *	MT928545 *	NA
*Lepidocyrtus* sp.	Brazil	MW193972	MW193972	NA	NA
*Lepidocyrtus* sp.	Costa Rica	CRBLB22488	**CRBLB22488-22**	NA	NA
*Lepidocyrtus* sp.	Costa Rica	CRBLB22501	**CRBLB22501-22**	NA	NA
*Lepidocyrtus* sp.	Costa Rica	CRBLB22506	**CRBLB22506-22**	NA	NA
*Lepidocyrtus* sp.	Costa Rica	CRBLB22524	**CRBLB22524-22**	NA	NA
*Lepidocyrtus* sp.	Costa Rica	CRBLB28162	**CRBLB28162-22**	NA	NA
*Lepidocyrtus* sp.	USA	OR169187	OR169187	NA	NA
*Lepidocyrtus* sp.	USA	OR169360	OR169360	NA	NA
*Lepidocyrtus* sp.	USA	OR169405	OR169405	NA	NA
*Lepidocyrtus* sp.	USA	OR169427	OR169427	NA	NA
*Lepidocyrtus* sp.	USA	OR169467	OR169467	NA	NA
*Lepidocyrtus* sp.	USA	OR169506	OR169506	NA	NA
*Lepidocyrtus* sp.	USA	OR170696	OR170696	NA	NA
*Lepidocyrtus* sp.	USA	OR172868	OR172868	NA	NA
*Lepidocyrtus* sp.	USA	OR172884	OR172884	NA	NA
*Lepidocyrtus* sp.	USA	OR172930	OR172930	NA	NA
*Lepidocyrtus* sp.	USA	OR172951	OR172951	NA	NA
*Lepidocyrtus* sp.	USA	OR173113	OR173113	NA	NA
*Lepidocyrtus* sp.	USA	OR174639	OR174639	NA	NA
*Lepidocyrtus vexans*	Panama	vePL64	NA	KF364904	KF364806
*Lepidocyrtus vexans*	Panama	vePL66	NA	KF364906	KF364808
*Lepidocyrtus vexans*	Panama	vePL67	NA	KF364907	KF364809
*Lepidocyrtus vexans*	Panama	vePL68	NA	KF364908	KF364810
*Seira* sp.	Panama	MH579503	MH579503	NA	NA
*Seira* sp.	Panama	MH579504	MH579504	NA	NA
*Seira* sp.	Panama	MH579505	MH579505	NA	NA
*Seira* sp.	Panama	FR28	NA	KF364849	KF364742
*Seira* sp.	Panama	FR48	NA	KF364850	KF364741
*Seira* sp.	Panama	FR95	NA	KF364851	KF364744

**Table 3:** Pseudopore distribution summary for *L.* cf. *nigrosetosus* and *L. olena*. The table displays the region and position of the body being evaluated and the number of pseudopores in that region for *L.* cf. *nigrosetosus* and *L. olena*.

Body Region	Position	*L.* cf. *nigrosetosus*	*L. olena*
Legs	Cx.I	2	2
	Cx.II	3	3
	Cx.III	2	2
Head dorsal	Pre-ocular	1	1
	Clypus	2	2
Body dorsal	Th.II (mid-dorsal)	1	1
	Th.III (mid-dorsal)	1	1
	Abd.I (mid-dorsal)	1	1
	Abd.II (mid-dorsal)	1	1
	Abd.III (mid-dorsal)	1	1
	Abd.IV (mid-dorsal)	1	1
	Abd.IV (dorsal)	0	0
Furca dorsal	Manubrial base	1	1
	Manubrial plate	2	2
	Dentes	4	2
Antenae ventral	Ant.III	1	1
	Ant.II	2	1
	Ant.I	0	0
Body ventral	Th.I	1	1
	Th.II	1	1
	Th.III	1	1
	Abd.I–VT base anterior	1	1
	Abd.I–VT base posterior	1	1
	Abd.II	1	1
	Abd.III–post-retinaculum	2	2
	Abd.IV	0	0
	Abd.V–pre-genital plate	1	1
Body lateral	Th.II	5	0
	Th.III	3–4	0
	Abd.I	0	0
	Abd.II	2–3	0
	Abd.III	4	0
	Abd.IV	0	0
	BP4	17	5–8

## Data Availability

The study’s molecular data has been deposited into the public database GenBank, and morphological data are provided as Tables and Figures.
